# Noninvasive ventilation in critically ill patients with the Middle East respiratory syndrome

**DOI:** 10.1111/irv.12635

**Published:** 2019-03-18

**Authors:** Basem M. Alraddadi, Ismael Qushmaq, Fahad M. Al‐Hameed, Yasser Mandourah, Ghaleb A. Almekhlafi, Jesna Jose, Awad Al‐Omari, Ayman Kharaba, Abdullah Almotairi, Kasim Al Khatib, Sarah Shalhoub, Ahmed Abdulmomen, Ahmed Mady, Othman Solaiman, Abdulsalam M. Al‐Aithan, Rajaa Al‐Raddadi, Ahmed Ragab, Hanan H. Balkhy, Abdulrahman Al Harthy, Musharaf Sadat, Haytham Tlayjeh, Laura Merson, Frederick G. Hayden, Robert A. Fowler, Yaseen M. Arabi, The Saudi Critical Care Trials Group

**Affiliations:** ^1^ Department of Medicine King Faisal Specialist Hospital and Research Center Jeddah Saudi Arabia; ^2^ Department of Medicine University of Jeddah Jeddah Saudi Arabia; ^3^ Department of Intensive Care College of Medicine, King Saud bin Abdulaziz University for Health Sciences, King Abdullah International Medical Research Center, King Abdulaziz Medical City Jeddah Saudi Arabia; ^4^ Prince Sultan Military Medical City Military Medical Services, Ministry of Defense Riyadh Saudi Arabia; ^5^ Department of Biostatistics and Bioinformatics, King Abdullah International Medical Research Center, College of Medicine King Saud bin Abdulaziz University for Health Sciences Riyadh Saudi Arabia; ^6^ Department of Intensive Care, Dr. Sulaiman Al‐Habib Group Hospitals, College of Medicine Alfaisal University Riyadh Saudi Arabia; ^7^ Department of Critical Care, Ohoud Hospitals King Fahad Hospital Al‐Madinah Al‐Monawarah Saudi Arabia; ^8^ Critical Care Medicine King Fahad Medical City Riyadh Saudi Arabia; ^9^ Intensive Care Department Al‐Noor Specialist Hospital Makkah Saudi Arabia; ^10^ Department of Medicine Division of Infectious Diseases, University of Western Ontario London Canada; ^11^ Department of Medicine Division of Infectious Diseases, King Fahad Armed Forces Hospital Jeddah Saudi Arabia; ^12^ King Saud University Riyadh Saudi Arabia; ^13^ Department of Anesthesiology, Intensive Care Tanta University Hospitals Tanta Egypt; ^14^ Intensive Care Department King Saud Medical City Riyadh Saudi Arabia; ^15^ King Faisal Specialist Hospital and Research Center Riyadh Saudi Arabia; ^16^ Intensive Care Department King Abdulaziz Hospital Al Ahsa Saudi Arabia; ^17^ Department of Family and Community Medicine King Abdulaziz University Hospital, Ministry of Health Jeddah Saudi Arabia; ^18^ Intensive Care Department King Fahd Hospital Jeddah Saudi Arabia; ^19^ Infection Prevention and Control Department, King Abdullah International Medical Research Center, College of Medicine King Abdulaziz Medical City, King Saud bin Abdulaziz University for Health Sciences Riyadh Saudi Arabia; ^20^ Intensive Care Department, King Abdullah International Medical Research Center, College of Medicine King Abdulaziz Medical City, King Saud bin Abdulaziz University for Health Sciences Riyadh Saudi Arabia; ^21^ Infectious Diseases Data Observatory, Churchill Hospital Oxford University, International Severe Acute Respiratory and Emerging Infection Consortium (ISARIC) Headington UK; ^22^ Department of Medicine, Division of Infectious Diseases and International Health University of Virginia School of Medicine, International Severe Acute Respiratory and Emerging Infection Consortium (ISARIC) Charlottesville Virginia; ^23^ Department of Critical Care Medicine and Department of Medicine, Sunnybrook Hospital, Institute of Health Policy Management and Evaluation University of Toronto Toronto Canada

**Keywords:** acute respiratory distress syndrome, coronavirus, Middle East respiratory syndrome, noninvasive ventilation, pneumonia, Saudi Arabia, severe acute respiratory infection

## Abstract

**Background:**

Noninvasive ventilation (NIV) has been used in patients with the Middle East respiratory syndrome (MERS) with acute hypoxemic respiratory failure, but the effectiveness of this approach has not been studied.

**Methods:**

Patients with MERS from 14 Saudi Arabian centers were included in this analysis. Patients who were initially managed with NIV were compared to patients who were managed only with invasive mechanical ventilation (invasive MV).

**Results:**

Of 302 MERS critically ill patients, NIV was used initially in 105 (35%) patients, whereas 197 (65%) patients were only managed with invasive MV. Patients who were managed with NIV initially had lower baseline SOFA score and less extensive infiltrates on chest radiograph compared with patients managed with invasive MV. The vast majority (92.4%) of patients who were managed initially with NIV required intubation and invasive mechanical ventilation, and were more likely to require inhaled nitric oxide compared to those who were managed initially with invasive MV. ICU and hospital length of stay were similar between NIV patients and invasive MV patients. The use of NIV was not independently associated with 90‐day mortality (propensity score‐adjusted odds ratio 0.61, 95% CI [0.23, 1.60] *P* = 0.27).

**Conclusions:**

In patients with MERS and acute hypoxemic respiratory failure, NIV failure was very high. The use of NIV was not associated with improved outcomes.

## 1 INTRODUCTION

Middle East respiratory syndrome (MERS) has emerged as a cause of severe respiratory illness in humans.^1^, ^2^ As of March 1, 2019, 2279 cases of MERS have been reported including 806 deaths.^3^ The disease presentation ranges from asymptomatic infection to severe respiratory illness, multiorgan failure, and death.^4^, ^5^, ^6^ Acute hypoxemic respiratory failure (AHRF) develops in up to 70% of hospitalized patients with MERS and is associated with high mortality.^7^, ^8^ To date, there is no specific antiviral therapy for MERS of proven effectiveness; supportive therapy remains the cornerstone of management.

Noninvasive ventilation has been increasingly used in the management of AHRF with variable success.^9^, ^10^, ^11^, ^12^ While NIV may initially avoid the need for intubation and invasive mechanical ventilation (MV), several studies have reported high failure rates and the need for invasive ventilation among patients with severe acute respiratory distress syndrome (ARDS) and an association with increased mortality.^12^ In a recent analysis from the LUNG SAFE study on unselected patients with ARDS, NIV was associated with higher intensive care unit (ICU) mortality in patients with the ratio of partial pressure of oxygen to the fraction of inspired oxygen (PaO_2_/FiO_2_) lower than 150 mm Hg.^12^ The role of NIV in AHRF secondary to viral respiratory infections is unclear. Although some uncontrolled studies suggested that NIV was effective and safe in management of patients with severe acute respiratory syndrome (SARS),^13^, ^14^, ^15^ others have highlighted concern of increased transmission risk to healthcare workers when patients with SARS are treated with NIV.^16^ Use of NIV in AHRF caused by pandemic H1N12009 virus (pdmH1N1) infection has been reported from several countries,^17^, ^18^, ^19^ with reported NIV failure reaching up to 85%.^17^ All studies were limited by their retrospective nature and, often, small sample size.

Noninvasive ventilation has been used in patients with MERS,^5^, ^8^ but its value in preventing intubation and impact on clinical outcomes has not been studied. The objective of this study was to assess the success of NIV in MERS patients with AHRF in avoiding intubation and its association with mortality and ICU and hospital length of stay. Our secondary objective was to identify factors associated with NIV failure in MERS patients.

## 2 METHODS

### 2.1 Study design and setting

We conducted this analysis on a multicenter retrospective cohort of critically ill MERS patients from 14 participating tertiary care hospitals in 5 cities in Saudi Arabia admitted between September 2012 and October 2015. The institutional review boards of all participating centers approved the study, and informed consent was not required due to the observational nature of the study. Details of the description of the cohort have been described before.^20^ Laboratory‐confirmed MERS was defined by the presence of positive real‐time reverse transcription‐polymerase chain reaction (rRT‐PCR) in upper or lower respiratory specimens.^21^

### 2.2 Definitions

In this study, we included all patients with AHRF who required mechanical ventilation support in the ICU, whether invasively or noninvasively. All patients who were managed initially with NIV were compared to those who were managed with invasive MV without NIV.

### 2.3 Data collection

For this analysis, we extracted baseline data including demographics, comorbidities, duration from onset of symptoms to emergency room admission, ICU admission, and intubation. Arterial blood gases, severity of illness measured by sequential organ failure assessment (SOFA), laboratory, and radiographic findings were collected on days 1, 3, 7, and 14 of ICU admission. The primary outcome was 90‐day mortality. ICU and hospital length of stay were collected. Duration of noninvasive and invasive mechanical ventilation and ventilator‐free days (based on 28‐day observation) was also calculated. We compared the two groups for the use of oxygen rescue therapies including neuromuscular blockade, high‐frequency oscillation ventilation, extracorporeal membrane oxygenation (ECMO), nitric oxide, and prone positioning.

### 2.4 Statistical analysis

Continuous variables were described as medians and interquartile ranges (Q1, Q3) or means and standard deviations and were tested using Mann‐Whitney *U* or Student's *t* test as appropriate. Categorical variables were reported as frequencies and proportions and tested using the chi‐square test or Fisher's exact test.

We compared NIV patients and invasive MV patients for baseline characteristics, collected variables during the ICU course, and measured clinical outcomes. Kaplan‐Meier curves censored at 90 days are plotted, and log‐rank test was used to compare survival time between patients treated with NIV and invasive MV.

Because of imbalances in baseline characteristics of NIV patients and invasive MV patients, we developed a propensity score for being treated with NIV with the following covariates used in the propensity score model development: age, SOFA score at admission to ICU, chronic pulmonary disease, chronic cardiac disease, chronic neurological disease, diabetes with chronic complications, PaCO_2_, PAO_2_:FiO_2_ ratio, and Glasgow Coma Scale. To examine the ability of the propensity score for accounting for the baseline characteristics, we carried out propensity score adjustment for the comparisons of baseline characteristics. We assessed the independent association of NIV with 90‐day mortality by multivariate logistic regression model adjusting for propensity score and clustering by centers. We also assessed the association of NIV with 90‐day mortality in subgroups of patients with PaO_2_/FiO_2_ ratio ≤100 and >100, and tested for interaction.

We performed a secondary comparison of patients who had failed NIV to those who had been successfully treated with NIV. All statistical tests were two‐sided with significance set at *α* < 0.05. Analyses were conducted using sas version 9.2 (SAS Institute, Cary, NC).

## 3 RESULTS

### 3.1 Baseline characteristics of patients treated with NIV and invasive MV

Of 330 critically ill MERS patients, 302 (89%) patients required ventilatory support and were included in our analysis. NIV was used as the initial ventilatory mode in 105/302 (34.8%) patients and invasive MV as the initial ventilatory mode in 197/302 (65%) patients.

Demographic and baseline characteristics of critically ill patients with MERS infection who required NIV compared to invasive MV are presented in Table 1. On ICU day 1, patients initially instituted on NIV were likely to have a lower SOFA score at baseline compared with patients who required invasive MV (median [Q1, Q3], 7 [4, 9] compared with 9 [7, 12] *P* < 0.0001). Median number of quadrants with infiltrates on chest radiograph was significantly lower among NIV patients compared with invasive MV patients, (2 [1, 4] compared with 3 [2, 4] *P* = 0.002). Median GCS was significantly higher among NIV patients compared with invasive MV patients (14 [5, 15], compared with 6 [3, 13] *P* < 0.0001). The ratio of partial pressure of oxygen in arterial blood to the fraction of inspired oxygen (PaO_2_/FiO_2_ ratio) on ICU day 1 was not different between NIV group and invasive MV group (110 [62, 160] compared to 106 [68, 166] with *P* = 0.56). Other physiological parameters on day 1 of admission to ICU are presented in Table 1. When adjusted for propensity score, the differences in most baseline characteristics were insignificant.

**Table 1:** Baseline characteristics of the study patients with Middle East respiratory syndrome (MERS) based upon initial treatment with noninvasive ventilation (NIV) compared to only treatment with invasive mechanical ventilation (invasive MV). Crude *P*‐values and propensity score‐adjusted *P*‐values are reported

Variables	NIV N = 105	Invasive MV N = 197	*P*‐value	Propensity score‐adjusted *P*‐value
Demographics				
Age (y)—Median (Q1, Q3)	60 (50, 73)	58 (45, 69)	0.09	>0.99
Body mass index (kg/m^2^)—Median (Q1, Q3)	28.9 (24.3, 34.5)	28.7 (24.2, 33.5)	0.88	0.73
Male sex—no. (%)	69 (65.7)	140 (71.1)	0.34	0.51
Healthcare‐associated, non‐healthcare worker—no.(%)	38 (36.2)	82 (41.6)	0.44	0.31
Healthcare worker—no. (%)	12 (11.4)	15 (7.6)		
Community‐acquired—no. (%)	55 (52.4)	100 (50.8)		
Days from onset of symptoms to the emergency room—Median (Q1, Q3)	5 (3, 8)	5 (3, 8)	0.76	0.31
Days from onset of symptoms to ICU admission—Median (Q1, Q3)	7 (5, 11)	7 (5, 11)	0.78	0.89
Days from onset of symptoms to intubation—Median (Q1, Q3)	8 (5, 12)	8 (5, 12)	0.32	0.58
Comorbidities—no. (%)				
Any comorbidity	88 (83.8)	164 (83.2)	0.90	0.35
Diabetes with chronic complications	62 (59.0)	95 (48.2)	0.07	>0.99
Chronic pulmonary disease (including asthma)	19 (18.1)	21 (10.7)	0.07	0.97
Chronic liver disease	8 (7.6)	11 (5.6)	0.49	0.32
Chronic renal disease	31 (29.5)	68 (34.5)	0.38	0.91
Chronic cardiac disease	50 (47.6)	78 (39.6)	0.18	>0.99
Chronic neurological disease	16 (15.2)	17 (8.6)	0.08	>0.99
Rheumatological disease	2 (1.9)	5 (2.5)	>0.99^a^	0.62
Any malignancy	10 (9.5)	21 (10.7)	0.76	0.74
Immunosuppressant use	5 (4.8)	15 (7.6)	0.34	0.87
Physiological parameters on ICU day 1—Median (Q1, Q3)				
PaO_2_(mm Hg)	63 (56, 74)	71 (60, 87)	0.003	0.15
FiO_2_	0.6 (0.5, 1)	0.7 (0.5, 1)	0.20	0.68
PaO_2_/FiO_2_ ratio	110 (62, 160)	106 (68, 166)	0.56	>0.99
PaCO2 (mm Hg)	39 (32, 47.4)	43.5 (36, 51.1)	0.027	>0.99
HCO_3_ mEq/L	23 (20, 24.7)	21.7 (18.1, 24.9)	0.30	0.62
Tidal volume (mL)	402 (350, 457)	400 (350, 435)	0.44	0.55
Tidal volume per kg of predicted body weight (mL/kg)	6.7 (5.8, 7.8)	6.6 (5.9, 7.6)	0.69	0.75
PEEP (cmH_2_0)	12 (8, 14)	12 (10, 14)	0.39	0.67
Plateau pressure (cmH_2_0)	28 (25, 30)	28 (22, 31)	0.38	0.33
Driving pressure (cmH_2_0)	17 (12, 18)	15 (12, 19)	0.76	0.99
Extra‐pulmonary parameters on ICU day 1—Median (Q1, Q3)				
Glasgow Coma Scale	14 (5, 15)	6 (3, 13)	<0.0001	>0.99
Mean arterial pressure (mm Hg)	70 (58, 83)	68 (60, 80)	0.90	0.05
Hemoglobin (g/dL)	11.1 (9.0, 13.2)	10.4 (8.5, 12.4)	0.19	0.86
Platelets (×10^9^/L)	176.5 (118.0, 254.0)	160.5 (96.0, 232.5)	0.10	0.73
Urine output (mL/24 h)	1120 (648, 1880)	1000 (365, 1800)	0.19	0.89
Bilirubin (µmol/L)	12.0 (6.8, 17.0)	12.0 (8.0, 24.4)	0.14	0.66
Creatinine (µmol/L)	115 (74, 211)	131 (75, 310)	0.16	0.29
Lactate (mmol/L)	1.6 (1.1, 2.3)	1.9 (1.2, 3.1)	0.06	0.25
International normalized ratio	1.1 (1, 1.3)	1.1 (1.0, 1.4)	0.31	0.28
Glucose (mmol/L)	10.7 (8.5, 13.4)	10.9 (7.7, 15.6)	0.99	0.48
Number of quadrants with infiltrates on chest radiograph	2 (1, 4)	3 (2, 4)	0.002	0.008
SOFA score—Median (Q1, Q3)	7 (4, 9)	9 (7, 12)	<0.0001	0.99
Respiratory SOFA score—Median (Q1, Q3)	2 (2, 2)	2 (2, 2)	0.37	0.54
Non‐respiratory SOFA score—Median (Q1, Q3)	5 (2, 7)	7 (5, 10)	<0.0001	0.93

FiO_2_, denotes the fraction of inspired oxygen; ICU, intensive care unit; Invasive MV, invasive mechanical ventilation; NIV, noninvasive ventilation; PaO_2_, partial pressure of oxygen in arterial blood; PaCO2, partial pressure of carbon dioxide in arterial blood; PEEP, positive end‐expiratory pressure; SOFA, sequential organ failure assessment;

The numbers of patients with missing data in noninvasive ventilation group and the invasive ventilation group, respectively, were as follows: Age—one patient and 0 patient; BMI—13 patients and 55 patients; Days from onset of symptoms to the emergency room—22 patients and 40 patients; Days from onset of symptoms to ICU admission—six patients and eight patient; Days from onset of symptoms to intubation—12 patients and nine patient; PaO2—two patients and one patients; PaO_2_/FiO_2_ ratio—three patients and six patients; PCO_2_—eight patients and one patients; HCO_3_—eight patients and 13 patients; Tidal volume—63 patients and 43 patients; PEEP—62 patients and 40 patients; Plateau pressure—87 patients and 118 patients; Driving pressure—87 patients and 118 patients; GCS—five patients and six patients; Mean arterial pressure—one patients and four patients; Hemoglobin—four patients and five patients; Platelets—one patients and five patients; Urine output—six patients and 14 patients; Bilirubin—two patients and nine patients; Creatinine—0 patients and three patients; lactate—28 patients and 52 patients; International normalized ratio—10 patients and 17 patients; Glucose—six patients and 21 patients; Number of quadrants with infiltrates on chest radiograph—18 patients and 24 patients; Respiratory SOFA score—three patients and six patients.

For continuous variables, Mann‐Whitney *U* test was used to calculate the *P* value. For categorical variables, chi‐square test was used to calculate the *P* value unless otherwise noted.

a Fisher's exact test was used to calculate *P* value.

### 3.2 Main interventions and outcomes

In 105 patients who were managed initially with NIV, NIV was used for a median duration of 1 (1, 3) day and 97 patients (92.4%) eventually required intubation and invasive MV (Table 2). Although SOFA score was lower among patients initially instituted on NIV compared with invasive MV, by day 14, the scores were similar in both groups (Figure S1). Patients managed initially with NIV were more likely to require nitric oxide subsequently compared to invasive MV patients [20.0% vs 11.7%, *P* = 0.05], although other oxygen rescue therapies were not different (Table 2).

**Table 2:** Main interventions in patients with Middle East respiratory syndrome (MERS) based upon initial treatment with noninvasive ventilation (NIV) compared to only treatment with invasive mechanical ventilation (invasive MV)

Variables	NIV N = 105	Invasive MV N = 197	*P*‐value
Invasive MV—no. (%)	97 (92.4)	197 (100.0)	0.0002^a^
Invasive MV duration—days			
Median (Q1, Q3)	8 (4, 18)	10 (5, 17)	0.25
Mean ± SD	12.5 ± 12.5	13.1 ± 12.5	0.25
Invasive MV‐free days (by day 28)			
Median (Q1, Q3)	0.0 (0.0, 12.0)	0.0 (0.0, 0.0)	<0.0001
Mean ± SD	6.8 ± 10.7	2.7 ± 6.7	<0.0001
NIV duration—days			
Median (Q1, Q3)	1.0 (1.0, 3.0)	—	
Mean ± SD	2.7 ± 4.2		
Total NIV and invasive MV duration—days			
Median (Q1, Q3)	10.0 (5.0, 20.0)	10.0 (5.0, 17.0)	0.70
Mean ± SD	14.3 ± 13.4	13.3 ± 12.5	0.70
Total NIV and invasive MV‐free days (by day 28)			
Median (Q1, Q3)	0.0 (0.0, 10.0)	0.0 (0.0, 0.0)	0.004
Mean ± SD	5.8 ± 9.7	2.7 ± 6.7	0.004
Vasopressor therapy—no. (%)	88 (83.8)	177 (89.8)	0.13
Renal replacement therapy—no. (%)	49 (46.7)	115 (58.4)	0.05
Duration	6.0 (3.0, 15.0)	8.0 (4.0, 14.0)	0.68
Oxygen rescue therapy			
Neuromuscular blockade—no. (%)	51 (48.6)	82 (41.6)	0.25
High‐frequency oscillation ventilation—no. (%)	9 (8.6)	17 (8.6)	0.99
ECMO—no. (%)	11 (10.5)	11 (5.6)	0.12
Nitric oxide—no. (%)	21 (20.0)	23 (11.7)	0.05
Prone positioning—no. (%)	12 (11.4)	21 (10.7)	0.84
Any oxygen rescue therapy—no. (%)	58 (55.2)	99 (50.3)	0.41

ECMO, extracorporeal membrane oxygenation; Invasive MV, invasive mechanical ventilation; NIV, noninvasive ventilation.

The numbers of patients with missing data in noninvasive ventilation group and the invasive ventilation group, respectively, were as follows: Invasive MV duration—11 patient and five patients; Noninvasive ventilation duration—four patient and 0 patients; Total NIV and invasive MV duration—five patient and 0 patients; NIV and invasive ventilation‐free days (by day 28)—one patient and 0 patients; Renal replacement therapy duration—66 patient and 106 patients; Denominator of the percentage is the total number of subjects in the group; For continuous variables, the Mann‐Whitney *U* test was used to calculate *P* value; For categorical variables, chi‐square test was used to calculate the *P* value unless otherwise noted.

a Fisher's exact test was used to calculate *P* value.

**Figure 1:**
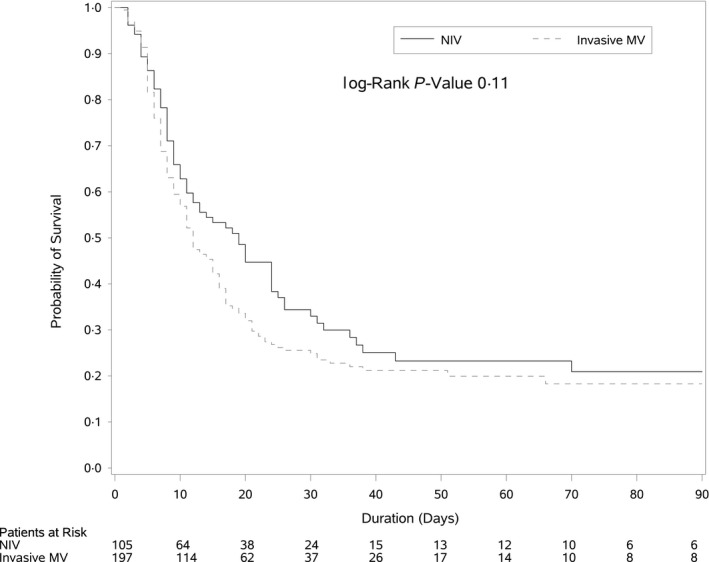
Kaplan‐Meier plot of cumulative survival for patients with MERS based upon initial treatment with noninvasive ventilation (NIV) compared to only treatment with invasive mechanical ventilation (invasive MV)

Crude 90‐day mortality was lower in the NIV group (69/105 [65.7%] compared to 150/197 [76.1%], *P* = 0.05, Table 3). After adjustment using propensity score, NIV was not associated with mortality (OR 0.61, 95% CI [0.23, 1.60] *P* = 0.27). Survival analysis of 90 days showed no difference in mortality (Figure 1). There was no significant association of NIV with 90‐day mortality in subgroups of patients with PaO_2_/FiO_2_ ratio ≤100 and >100 [OR 0.56, 95% CI 0.12, 2.66, *P* = 0.42 and OR 0.54, 95% CI 0.18, 1.61, *P* = 0.22, respectively; *P* value for interaction: 0.65] (Table 4). There were no between‐group differences in serial PaO_2_/FiO_2_ ratio, PCO_2_ over time Figure S1.

**Table 3:** Outcomes in patients with Middle East respiratory syndrome (MERS) based upon initial treatment with noninvasive ventilation (NIV) compared to only treatment with invasive mechanical ventilation (invasive MV)

Variables	NIV N = 105	Invasive MV N = 197	*P*‐value
Hospital mortality—no. (%)	70 (66.7)	150 (76.1)	0.08
90‐d mortality—no. (%)	69 (65.7)	150 (76.1)	0.05
ICU mortality—no. (%)	68 (64.8)	149 (75.6)	0.059
ICU length of stay, days—Median (Q1, Q3)	11 (6, 24)	11 (6, 18)	0.79
Hospital length of stay, days—Median (Q1, Q3)	22 (12, 38)	20 (11, 35)	0.60

ICU, intensive care unit; Invasive MV, invasive mechanical ventilation; NIV, noninvasive ventilation.

Denominator of the percentage is the total number of subjects in the group. For continuous variables, the chi‐square test was used to calculate *P* value. For continuous variables, Mann‐Whitney *U* test was used to calculate the *P* value. For categorical variables, chi‐square test was used to calculate the *P* value. The numbers of patients with missing data in noninvasive ventilation group and the invasive ventilation group, respectively, were as follows: ICU Mortality—1 patient and 0 patients; ICU length of stay—two patient and four patients

Median ICU and hospital length of stay were similar between NIV patients and invasive MV patients, (11 days [6, 24] compared to 11 days [6, 18], *P* = 0.79, and 22 days [12, 38] compared to 20 days [11, 35], *P* = 0.6). There was no significant difference in the duration of invasive MV and total duration of NIV and invasive MV between the two groups, although invasive MV‐free days and total NIV and invasive MV‐free days were significantly longer among NIV patients compared to invasive MV patients (Table 2, Figure S2).

### 3.3 Comparison of patients who failed NIV vs patients successfully treated only with NIV

Overall, only 8/105 (7.6%) of the NIV patients avoided subsequent intubation (Table S1). These patients were significantly younger than those who failed NIV (median age [Q1, Q3]: 45 years [35.5, 55.0] vs 61 years [52, 73.5], *P* = 0.007) and had much lower baseline SOFA score (median SOFA [Q1, Q3] 2.5 [2.0, 4.0] vs 7.0 [4.0, 9.0], *P* = 0.003; Tables S1 and S2). Crude 90‐day mortality was significantly higher in patients who failed NIV compared with patients successfully treated only with NIV (Table S3 and Figure S3).

## 4 DISCUSSION

We have shown that among patients with MERS‐related AHRF, NIV was commonly used, but nearly always resulted in subsequent transition to invasive ventilation. Our results suggest that while the initial NIV use in MERS patients was not associated with reduction of mortality or length of ICU or hospital stay, these patients had greater requirement for subsequent inhaled nitric oxide. A minority of patients were successfully managed with NIV—those who were young and had less severe disease. These findings have important implications for early management of patients infected with MERS, specifically, that there is little advantage to initial NIV treatment for most patients with MERS‐related AHRF and that NIV may be associated with greater subsequent need for oxygenation rescue therapy such as inhaled nitric oxide.

**Table 4:** Association between noninvasive ventilation (NIV) and 90‐d mortality among patients with the Middle East respiratory syndrome (MERS) after adjusting for propensity score

NIV vs No NIV^a^	Number of subjects	OR (95% CI)	*P*‐value	*P* value for interaction
All patients	267	0.61 (0.23, 1.60)	0.27	—
PaO_2_/FiO_2_ ratio ≤ 100	132	0.56 (0.12, 2.66)	0.42	0.65
PaO_2_/FiO_2_ ratio > 100	135	0.54 (0.18, 1.61)	0.22	

OR, odds ratio; CI, confidence interval.

Chi‐square test is used to calculate the *P*‐value.

a Adjusted for propensity score (which was calculated from age, SOFA, chronic cardiac disease, diabetes with chronic complications, chronic pulmonary disease, chronic neurological disease, PCO_2_ (mm Hg), PAO_2_/FiO_2_ ratio, and GCS) and clustering by centers

Noninvasive ventilation has been proven to be useful as a means to avoid intubation and improve clinical outcomes in certain conditions, generally, with the possibility for rather rapid reversal of respiratory failure—for example, pulmonary edema due to congestive heart failure, and respiratory failure due to COPD exacerbations.^22^, ^23^ For conditions that typically worsen or do not improve in the range of many hours (eg, most causes of pneumonia), there appears to be little advantage in using NIV as a means to avoid intubation.^9^, ^24^, ^25^ In choosing to use NIV for as initial treatment for patients with hypoxemic respiratory failure, there is a practical risk of patients worsening on NIV and requiring intubation at a time when they already have more advanced organ failure.

Few studies have assessed the effectiveness of NIV in patients with AHRF secondary to ARDS and acute lung injury. The overall effectiveness of NIV in reducing intubation rate or improving clinical outcome in these patients remains controversial.^26^, ^27^, ^28^ Post hoc analysis of the LUNG SAFE study found that NIV was used in 15% of patients with ARDS and was associated with higher ICU mortality in subset of patients with severe ARDS.^12^ A randomized controlled trial of patients with AHRF showed that NIV was not effective in reducing intubation rate compared to high flow and standard oxygen therapy, and was associated with higher mortality.^29^ However, there may be important differences in care and outcomes according to the NIV interface, for example, a nasal, nose and mouth, full face, or helmet device.^30^

NIV is generally not recommended for patients with hypoxia secondary to respiratory infections due to lack of efficacy and the potential for pathogen transmission.^16^, ^31^, ^32^ It is also considered one of the aerosol‐generating procedures that may increase risk of transmission to healthcare workers.^32^ Broad dispersion of exhaled air during NIV via a face mask has previously been shown using a simulated patient encounter.^33^ Although NIV was successfully used in a small number of SARS patients without documented nosocomial transmission,^13^ other studies showed that NIV might have led to increased nosocomial transmission.^16^, ^34^ In our cohort, NIV failure was strikingly high and was associated with more requirements for inhaled nitric oxide. However, it is important to note that the process responsible for higher requirement for inhaled nitric oxide in patients treated with NIV could be related to timing of transition from NIV to invasive mechanical ventilation (ie, if transition were delayed) rather than the use of NIV per se. Finally, the confidence interval around the point estimate for the association of initial NIV and 90‐day mortality is wide; therefore, a real difference cannot be entirely be excluded in either direction. However, given the very high failure rate, our data therefore indicate that use of NIV in patients with MERS should generally be avoided unless it was used for patients with the least severity of illness.

Our study has certain strengths. This is the largest cohort examining ventilation practices among critically ill patients with MERS that contains detailed demographic, baseline characteristics, physiology, interventions, and outcomes. Our study is also subject to several limitations. Due to the retrospective nature of the study, we do not have detailed settings of NIV (eg, PEEP and driving pressure), data on patient tolerance, the type of NIV mask interface (nasal or full‐face mask), pre‐ICU use of NIV, and reasons for intubation. We do not have data to address the association of NIV vs invasive ventilation with subsequent nosocomial pneumonia. Our study was not designed to address infection control issues related to NIV and risk of transmission to healthcare workers, and therefore, we cannot report on this outcome that is important to healthcare workers and other patients. Although we adjusted for imbalances in baseline characteristics using propensity score, the effect of residual unmeasured confounding cannot be excluded. Given the retrospective nature of our study, the potential effect of indication bias exists. For example, our data show that less severely ill patients as reflected by SOFA were more likely to have been managed by NIV. Nevertheless, we accounted for SOFA in the propensity score, and if residual bias exists, it would likely favor (show better outcomes with) NIV, making our findings a conservative estimate of any potential risks associated with NIV. In addition, the potential for lead‐time bias exists. Patients receiving NIV may have presented with less advanced disease, and the disease may have merely progressed while on NIV to the same point at which patients with more advanced had already presented and required invasive ventilation from outset. Due to limited number of patients who avoided intubation, we were not powered to identify independent predictors for NIV success.

In conclusion, we report the results of NIV use in MERS patients from a large cohort of critically ill patients. We observed that there is little advantage to initial NIV treatment for most patients with MERS‐related AHRF and that NIV may be associated with greater subsequent need for oxygen rescue therapy.

## CONFLICT OF INTEREST

The authors have no conflict of interest to disclose.

## AUTHOR CONTRIBUTIONS

BMA: Conception and design, data acquisition, analytical plan, interpretation of data for the work, drafting of the manuscript, critical revision of the manuscript for important intellectual content, approval of the final version to be published, and agreement to be accountable for all aspects of the work. IQ, FH, YM, GM, AO, AK, AA, KK, SS, AAM, AM, OS, AMA, RR, AR, HB, AH, MS, and HT: Data acquisition, critical revision of the manuscript for important intellectual content, approval of the final version to be published, and agreement to be accountable for all aspects of the work. JJ: Conception and design, analytical plan, data analysis, critical revision of the manuscript for important intellectual content, approval of the final version to be published, and agreement to be accountable for all aspects of the work. LM, FGH, RAF, and YA: Conception and design, analytical plan, interpretation of data for the work, critical revision of the manuscript for important intellectual content, approval of the final version to be published, and agreement to be accountable for all aspects of the work.
