# Supplementary material for: Noninvasive ventilation in critically ill patients with the Middle East respiratory syndrome
Source: Influenza Other Respir Viruses. 2019 Mar 18;13(4):382–90. doi: 10.1111/irv.12635 (PMC6586182; doi:10.1111/irv.12635)
Supplement: Supplementary file 1 [file IRV-13-382-s001.docx]

**Supplementary appendix to**

**Noninvasive Ventilation in Patients with the Middle East Respiratory Syndrome (MERS)**

**Collaborators**

| Center | Names |
| --- | --- |
| Saudi Arabia-The Saudi Critical Care Trial group |  |
| King Saud bin Abdulaziz University for Health Sciences and King Abdullah International Medical Research Center, Riyadh | Yaseen Arabi  Abdulaziz Aldawood  Hanan Balkhy  Mohamed A. Hussein  Mashael Al Ahmadi  Musharaf Sadat  Hanan Al Mutairi  Eman Al Qasim  Ahmed Deeb  Hasan Aldorzi  Jesna Jose  Mundekkadan Shihab  Sheryl Ann Abdukahil  Aron Toledo  Lara Afesh  Mohammed Rafique Sohail |
| King Fahad Medical City, Riyadh | Abdullah Al Motairi |
| Prince Sultan Cardiac Center, Riyadh | Ghaleb A. Almekhlafi  Yasser Mandourah  Sahar Hassan  Abid Alwan  Raylin Cabal  Rima E Mahamed  Khaloud M. Harbi  Abdulelah Ala Haidary |
| King Saud Medical City, Riyadh | Abelrahman Al-Harthy  Ahmed Fouad Mady   Omar Elsayed Ramadan  Muhammed Asim Rana  Basim Raafat Huwait  Mohamed Ali Al-Odat  Waleed Tharwat Al-Atreeby |
| King Faisal Specialist Hospital and Research Centre, Riyadh | Othman Solaiman |
| King Khalid University hospital, Riyadh | Ahmed Abdul Mommin  Muhammed Fares  Mazen Barry |
| Security Forces Hospital, Riyadh | Awad Al Omari |
| King Abdulaziz Medical City, Jeddah | Fahad Al-Hameed  Jalal Al Refai |
| King Fahd Armed Forced Hospital, Jeddah | Sarah Shalhoub |
| King Faisel Hospital Research Center, Jeddah | Basem M Alraddadi  Rashed E Alrehaili  Sarah Batawi |
| King Fahad Hospital Group, Jeddah | Anees Sindi  Rajaa Al-Raddadi  Ahmed Rajab  Omimah Shabouni  Abeer Mustafa Housa  Amal AbdulallaTurkistani  Abdullatif Ayesh Almarashi  Amaal Ali Sarraj  Salwa Awad Own  Sara Mohammed AlJeaid  Wijdan Abdulalkareem Baeshen |
| AlNoor Specialist Hospital, Makkah | Kasim Al Khatib  Hamdy Badr  Majduldeen Azzo |
| King Abdulaziz Medical city-Alahsa | Abdulsalam Alaithan |
|  |  |
| King Fahad Hospital, Madinah | Ayman Kharaba  Noah Noor |
| **United Kingdom** |  |
| Infectious Diseases Data Observatory, Oxford University | Laura Merson |
| Canada |  |
| Sunnybrook Health Sciences Centre, Canada | Robert Fowler  Ruxandra Pinto |
| United States of America |  |
| University of Virginia School of Medicine, USA | Frederick Hayden |
|  |  |

**eTable 1.** Baseline characteristics of patients with Middle East Respiratory Syndrome (MERS) with failed noninvasive ventilation (NIV) compared with those with successful NIV.

| **Variables** | **Failed NIV N=97** | **Successful NIV**  **N=8** | **Pvalue** |
| --- | --- | --- | --- |
| **Demographics** | | |  |
| Age (yr), Median (Q1,Q3) | 61.0 (52.0, 73.5) | 45.0 (35.5, 55.0) | 0.007* |
| BMI (kg/m2), Median (Q1,Q3) | 29.7 (24.3, 34.6) | 26.6 (20.1, 33.8) | 0.61* |
| Male sex – no. (%) | 61 (62.9) | 8 (100.0) | 0.05 |
|  |  |  |  |
| Community-acquired – no. (%) | 49 (50.5) | 6 (75.0) | 0.31 |
| Healthcare-associated, non healthcare worker – no. (%) | 37 (38.1) | 1 (12.5) |  |
| Healthcare worker – no. (%) | 11 (11.3) | 1 (12.5) |  |
|  |  |  |  |
| Days from onset of symptoms to the emergency room – Median (Q1,Q3) | 4.5 (3.0, 7.0) | 8.0 (4.0, 11.0) | 0.18 |
| Days from onset of symptoms to ICU admission – Median (Q1,Q3) | 7.0 (4.0, 11.0) | 8.0 (5.5, 10.5) | 0.71 |
| Days from onset of symptoms to intubation – Median (Q1,Q3) | 8.0 (5.0, 12.0) | - |  |
|  |  |  |  |
| **Comorbidities** – **no. (%)** |  |  |  |
| Any Comorbidities | 82 (84.5) | 6 (75.0) | 0.61 |
| Diabetes with chronic complications | 58 (59.8) | 4 (50.0) | 0.71 |
| Chronic pulmonary disease (including asthma) | 19 (19.6) | 0 (0) | 0.35 |
| Chronic liver disease | 8 (8.2) | 0 (0) | >0.99 |
| Chronic renal disease | 29 (29.9) | 2 (25.0) | >0.99 |
| Chronic cardiac disease | 48 (49.5) | 2 (25.0) | 0.27 |
| Chronic neurological disease | 16 (16.5) | 0 (0.0) | 0.60 |
| Rheumatological disease | 1 (1.1) | 1 (12.5) | 0.15 |
| Any malignancy | 10 (10.3) | 0 (0) | >0.99 |
| Immunosuppressant use | 4 (4.1) | 1 (12.5) | 0.33 |
|  |  |  |  |
| **Physiological parameters on ICU day 1** – Median(Q1,Q3) |  |  |  |
| PaO_2_ (mmHg) | 63.0 (56.2, 74.4) | 62.5 (56.5, 77.0) | 0.95 |
| FiO_2_ | 0.6 (0.5, 1.0) | 0.6 (0.4, 0.9) | 0.46 |
| PaO_2_/FiO_2_ ratio | 103 (62, 160) | 116 (77, 180) | 0.67 |
| PCO_2_ (mmHg) | 39.1 (31.3, 47.4) | 37.1 (32.3, 49.0) | 0.81 |
| HCO_3_ | 23.0 (19.7, 25.0) | 21.1 (21.0, 22.6) | 0.31 |
| Tidal volume (ml) | 401.5 (350.0, 457.0) | - |  |
| Tidal volume per kg of predicted body weight (ml/kg) | 6.7 (5.8, 7.8) | - |  |
| PEEP (cmH_2_0) | 12.0 (8.0, 14.0) | - |  |
| Plateau pressure (cmH_2_0) | 28.0 (25.0, 30.0) | - |  |
| Driving Pressure (cmH_2_0) | 17.0 (12.0, 18.0) | - |  |
|  |  |  |  |
| **Extra-pulmonary parameters on ICU day 1** – Median (Q1,Q3) |  |  |  |
| GCS | 12.0 (4.5, 15.0) | 15.0 (15.0, 15.0) | 0.01 |
| Mean Arterial Pressure (mmHg) | 69.0 (57.5, 80.0) | 83.5 (76.8, 86.5) | 0.02 |
| Haemoglobin (g/dL) | 11.0 (9.0, 13.2) | 12.2 (8.5, 13.7) | 0.68 |
| Platelets(x10^9^/L) | 176.5 (116.5, 259.0) | 175.5 (130.5, 212.0) | 0.90 |
| Urine output (mL/24 hours) | 1100.0 (640.0, 1790.0) | 1890.0 (1150.0, 2890.0) | 0.09 |
| Bilirubin (µmol/L) | 12.0 (6.9, 19.0) | 11.8 (5.0, 15.0) | 0.49 |
| Creatinine (µmol/L) | 126.0 (75.0, 211.0) | 69.0 (63.0, 194.0) | 0.11 |
| Lactate (mmol/L) | 1.5 (1.1, 2.4) | 2.0 (1.2, 2.1) | 0.80 |
| INR | 1.1 (1.0, 1.3) | 1.1 (1.0, 1.2) | 0.50 |
| Glucose (mmol/L) | 10.7 (8.5, 14.3) | 9.4 (6.4, 13.0) | 0.34 |
| Number of quadrants with infiltrates on chest radiograph | 2.0 (1.0, 4.0) | 4.0 (3.0, 4.0) | 0.08 |
|  |  |  |  |
| SOFA score – Median (Q1,Q3) | 7 (4, 9) | 3 (2, 4) | 0.003 |
| Respiratory SOFA score – Median (Q1,Q3) | 2 (2, 2) | 2 (2, 2) | 0.45 |
| Non-respiratory SOFA score – Median (Q1,Q3) | 5 (2, 8) | 1 (0, 2) | 0.002 |

BMI–Body mass index; FiO**_2_**: denotes the fraction of inspired oxygen, PaO**_2_**: partial pressure of oxygen in arterial blood, PaCO**_2_**: partial pressure of carbon dioxide, PEEP: positive end-expiratory pressure, GCS: Glasgow Coma Scale, ALT: Alanine amino transferase, INR: international normalized ratio, SOFA: Sequential Organ Failure Assessment. Mann-Whitney U test is used to calculate the Pvalue

For continuous variables, Mann-Whitney U test was used to calculate p value except for p values labeled with * sign indicating the use of t-test

For categorical variables, the Fishers exact test was used to calculate p value

**eTable 2.** Main interventions in patients with Middle East Respiratory Syndrome (MERS) with failed noninvasive ventilation (NIV) compared with those with successful NIV.

| **Variables** | **Failed NIV N=97** | **Successful NIV**  **N=8** | **P-value** |
| --- | --- | --- | --- |
| **Interventions** | | | |
| Non-invasive positive pressure ventilation – no. (%) | 97 (100.0 ) | 8 (100.0 ) |  |
| Duration – Median (Q1,Q3) | 1.0 (1.0, 3.0) | 2.5 (1.5, 5.5) | 0.07 |
| Invasive ventilation – no. (%) | 97 (100.0 ) | 0 (0) | <0.0001 |
| Duration – Median (Q1, Q3) | 8.0 (3.0, 17.0) | - |  |
| Vasopressors – no. (%) | 87 (89.7) | 1 (12.5) | <0.0001 |
| Renal replacement therapy – no. (%) | 48 (49.5) | 1 (12.5) | 0.06 |
| Duration | 6.0 (3.0, 15.0) | 13.0 (13.0, 13.0) | 0.62 |
| Neuromuscular blockade – no. (%) | 51 (52.6 ) | 0 (0) | 0.006 |
| High-frequency oscillation ventilation – no. (%) | 9 (9.3) | 0 (0) | >0.99 |
| ECMO – no. (%) | 11 (11.3) | 0 (0) | 0.60 |
| Nitric oxide – no. (%) | 21 (21.6) | 0 (0) | 0.35 |
| Prone positioning – no. (%) | 12 (12.4) | 0 (0) | 0.59 |
| Any oxygen rescue therapy – no. (%) | 58 (59.8) | 0 (0) | 0.001 |

**ECMO:** extracorporeal membrane oxygenation; **ICU:** Intensive care unit

For continuous variables, the Mann-Whitney U test was used to calculate p value

For categorical variables, the Fishers exact test  was used to calculate p value

| **Variables** | **Failed NIV N=97** | **Successful NIV**  **N=8** | **P-value** |
| --- | --- | --- | --- |
| Hospital mortality – no. (%) | 70 (72.2) | 0 (0) | <0.0001 |
| 90-day mortality – no. (%) | 69 (71.1) | 0 (0) | 0.0001 |
| 28-day mortality – no. (%) | 61 (62.9) | 0 (0) | 0.0006 |
| 14-day mortality – no. (%) | 45 (46.4) | 0 (0) | 0.01 |
| ICU mortality – no. (%) | 68 (70.1) | 0 (0) | 0.0001 |
| ICU length of stay, days – Median (Q1,Q3) | 11.0 (6.0, 24.0) | 7.0 (3.5, 11.0) | 0.11 |
| Hospital length of stay, days – Median (Q1,Q3) | 22.0 (12.0, 38.0) | 21.0 (14.5, 50.0) | 0.57 |

**eTable 3**. Outcomes of patients with Middle East Respiratory Syndrome (MERS) with failed noninvasive ventilation (NIV) compared with those with successful NIV.

**eFigure 1:** Comparison of serial ratio of partial pressure of oxygen to the fraction of inspired oxygen (PaO_2_/FiO_2_ ratio), partial pressure of carbon dioxide(PCO_2_) and Sequential Organ Failure Assessment (SOFA) scores between patients who were managed initially by noninvasive ventilation (NIV) compared to invasive mechanical ventilation (invasive MV).

| 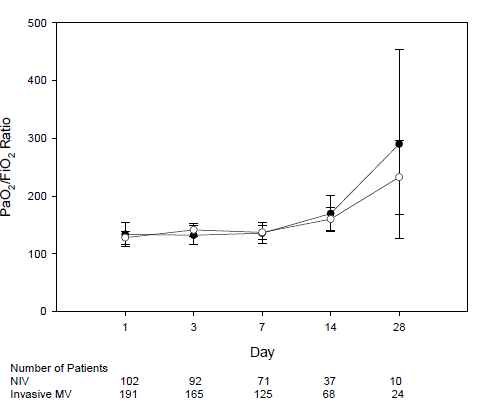P= 0.70 for between-group differences  P= 0.60 for between-group differences over time | 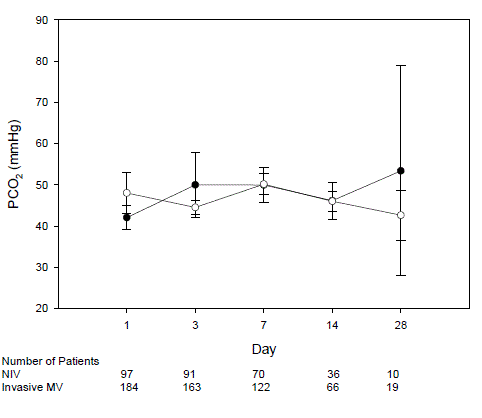P= 0.008 for between-group differences  P= 0.20 for between-group differences over time |
| --- | --- |
| 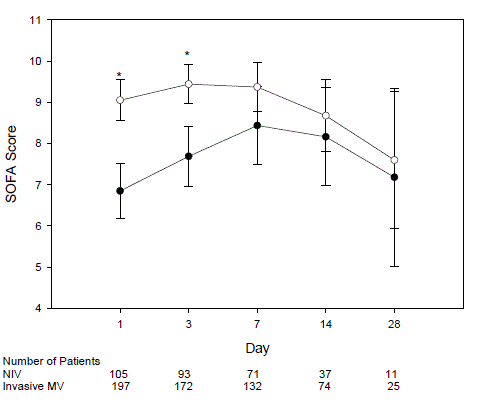P= 0.04 for between-group differences  P= 0.001 for between-group differences over time |  |
| 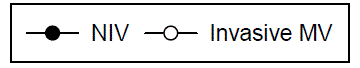 | |

**eFigure 2:** Distribution of invasive ventilation duration between patients who were managed initially by NIV and invasive NIV

**
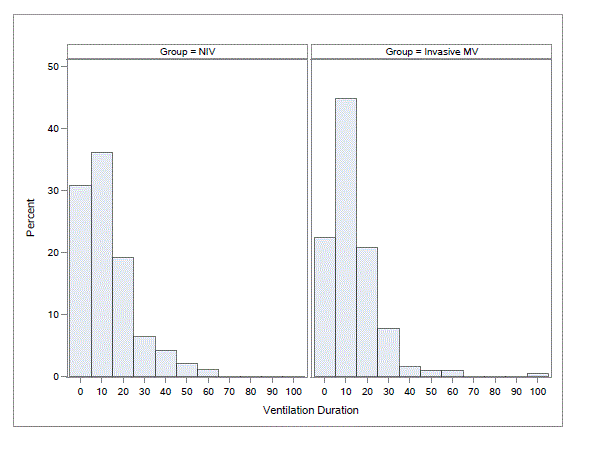
eFigure 3:** Distribution of invasive ventilation duration between NIV and invasive NIV among 90-day non-survivors (Panel A) and survivors (Panel B)

| **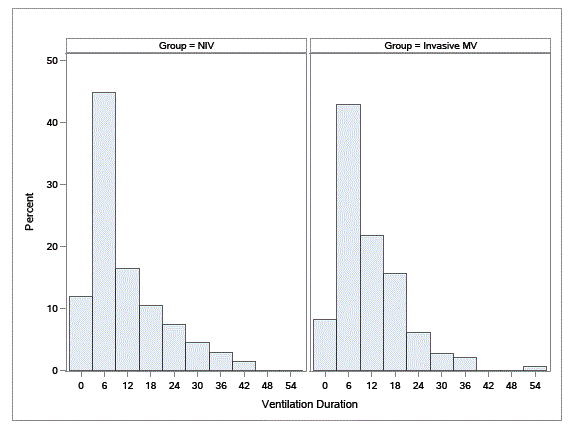A** |
| --- |
| **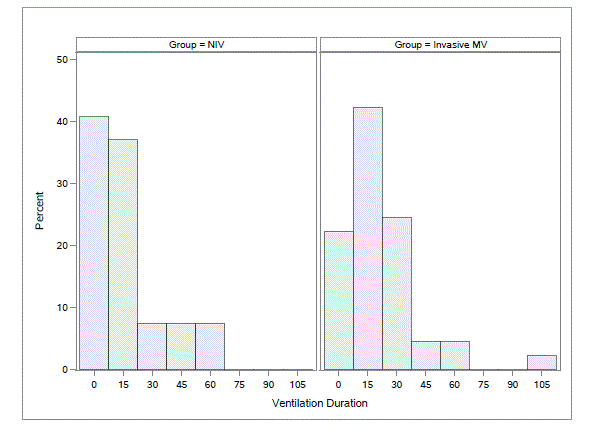B** |
